# Challenges of determining the relative contribution of determinants of health on population health: a Canadian perspective

**DOI:** 10.1177/17579139241236655

**Published:** 2024-05-17

**Authors:** M Aloosh, J Hopkins

**Affiliations:** Department of Health Research Methods, Evidence, and Impact, Michael G. Degroote School of Medicine, McMaster University, 100 Main St W, Hamilton, ON L8P 1H6, Canada; Department of Health Research Methods, Evidence, and Impact, Michael G. Degroote School of Medicine, McMaster University, Hamilton, ON, Canada; Public Health Ontario, Toronto, ON, Canada

## Abstract

This article investigates the source of frequently cited data regarding the relative contribution of determinants of health to population health in Canada. It critically discusses the imperative for such national or regional data in policymaking, and the challenges and limitations of this approach.

The use of evidence in public health decision-making is intended to increase the likelihood of achieving better health outcomes and reducing unintended negative consequences on health. The currency of evidence is also critical because constructs and indicators can change over time. Using outdated or inaccurate information can lead to inappropriate or ineffective interventions, exacerbating the situation. Previously, it has been found that errors in the literature can be perpetuated over time.^1^ Therefore, critical appraisal and periodic review of evidence are essential to ensure that public health decisions are informed by timely and reliable evidence.

The COVID-19 pandemic has highlighted the importance of relying on current evidence in making public health decisions. For instance, it has been observed that Black communities have been disproportionately affected by COVID-19. In Canada, COVID-19-related mortality among Black people was 2.2 times higher than that of White and non-Indigenous people.^2^ In addition, individuals from Black communities exhibited a lower vaccination rate (56.4%) compared to non-visible minority individuals (77.7%) and the South Asian population (82.5%).^3^ In response, public health agencies have taken steps to encourage vaccination in Black communities.^4^ However, recent evidence showed that vaccine hesitancy in Black communities stems from mistrust and structural racism, beyond just misinformation or health literacy gaps.^5^

**Figure:**
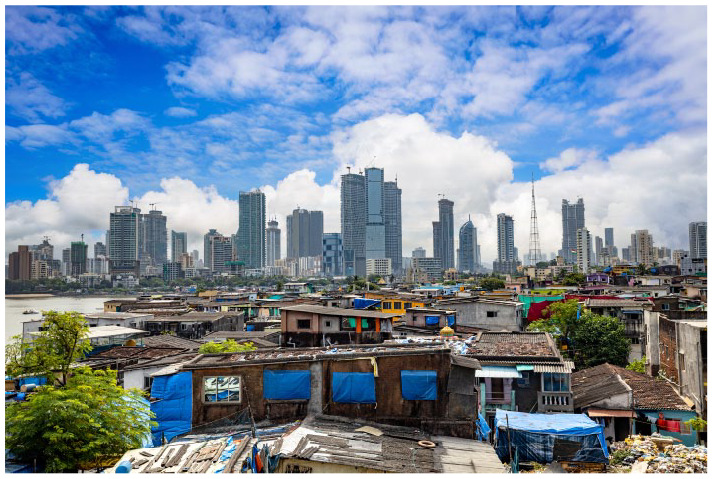


Recently, we investigated the source of frequently cited estimates regarding the relative contribution of determinants of health on Canadians’ health. The estimates show that health in Canada is affected by social determinants of health (50%), healthcare (25%), biology (15%), and the environment (10%).^6^ These estimates have been cited broadly in the international public health community and used in many presentations, theses, manuscripts,^7–9^ reports,^6,10–12^ and World Health Organization documents.^13^ Several of these documents cited a 2012 document from the Canadian Institute of Advanced Research (CIAR),^7,9,13^ a Canadian Medical Association report from 2013,^8^ a report from a Canadian Senate committee from 2008,^11^ and a CIAR document from 2002.^10^ The oldest document we discovered contained estimates published in 2001 and referred to a CIAR document.^6^ Despite further investigation, we could not locate any document authored by CIAR containing these estimates and could not find information on the methodology for estimate calculations. Given the lack of transparency in sources and methods, we believe these estimates should no longer be cited or used as a source of evidence.

Addressing the absence of national estimates, exploring estimates of the relative contribution of determinants of health from other countries could seem to be a viable approach. Nonetheless, there is a scarcity of publications that delve into the relative impact of determinants of health on overarching health outcomes despite existing literature highlighting the significance of individual determinants of health.^14^ Even if an estimate were available, numerous challenges would still need to be navigated. First, the context of countries, such as their healthcare system structure and funding, differs, making it difficult to compare the contribution of healthcare to overall population health. Second, there are variations in the understanding of determinants of health and different categories of social determinants of health are defined differently in published reports. Third, the specific measures or indicators used within each category of determinants of health can vary significantly.^15^ That said, adopting the estimates of the relative contribution of determinants of health on health from another nation is not feasible.

Furthermore, additional complexities exist in relation to the use of a numerical estimate of the effect of determinants of health on health. Nonetheless, a more nuanced understanding of the determinants of health acknowledges that health is a product of multiple factors that interact in dynamic ways. An intersectional approach considers the interaction between various social identities and their associated determinants. It highlights the need to address the interconnected nature of social inequalities to achieve meaningful improvements in public health and health equity. This perspective challenges the idea of partitioning health outcomes and attributing them to a single determinant. Instead, it acknowledges that health disparities and outcomes emerge from a web of interconnected factors within a larger societal context.^16^

Intersectionality emphasizes that individuals have multiple social identities (e.g. race, gender, class, and sexuality) that intersect and interact to shape their experiences and opportunities. Each of these identities can impact health outcomes independently and in combination with others (e.g. being Black, a woman and transgender) with complex interactions which can also be cumulative.^17^ The impact of determinants can change over time and in different contexts. What affects one’s health at a particular point in life or in a place may differ from other times and places. An intersectoral approach provides a more holistic understanding of health disparities by recognizing that inequities result from systemic and structural factors.

Despite the limitations of the data and methods, understanding the relative contribution of determinants of health on overall health outcomes is essential for informing public health policies and interventions across diverse populations and contexts. While there are commonalities in the determinants that influence health outcomes globally, the specific contributions of each determinant can vary significantly based on social, economic, cultural, environmental, and healthcare factors unique to each country or region. Knowing which determinants of health have the most significant impact on health outcomes can help prioritize and allocate resources for the most effective interventions to improve health.^18^ Such a model satisfies policymakers’ need for benchmarks for prioritization and measuring progression toward defined goals.

Overall, the social determinants of the health framework remains relevant to how we conceptualize fairness and health.^19^ To enable action, we advocate for a comprehensive understanding of the relative contribution of determinants of health to broad health outcomes, specific to a country or region. This understanding can enable the development of effective interventions, resource allocation, and reducing health disparities. At the same time, it is essential to acknowledge the limitations of this approach. As George Box described, ‘all models are wrong, some are useful’.^20^
